# An EGFR Co-Amplified and De Novo Long Noncoding RNA HELDR Promotes Glioblastoma Malignancy through KAT7-Driven Gene Programs

**DOI:** 10.21203/rs.3.rs-6456987/v1

**Published:** 2025-06-24

**Authors:** Shi-Yuan Cheng, Xiaozhou Yu, Xiao Song, Richard Schäfer, Qingshu Meng, Deanna Tiek, Runxin Wu, Qiu He, Maya Walker, Rendong Yang, Qi Cao, Bo Hu

**Affiliations:** Northwestern University; Northwestern University; Northwestern University; Northwestern Univeristy; Department of Urology, Feinberg School of Medicine, Northwestern University; Northwestern University Feinberg School of Medicine; Northwestern Univeristy; Northwestern Univeristy; Northwestern Univeristy; Northwestern University; Northwestern University; Northwestern University Feinberg School of Medicine

**Keywords:** Glioblastoma, EGFR, ELDR, lncRNA, KAT7

## Abstract

EGFR amplification frequently happens within extrachromosome DNAs (ecDNAs) and is a major mutation in glioblastoma (GBM). However, targeting EGFR for GBM treatments has been unsuccessful. Here we characterized a long non-coding RNA (lncRNA) that is co-amplified with EGFR within ecDNAs that we name hidden EGFR long non-coding downstream RNA (HELDR). HELDR is a GBM-specific lncRNA that promotes tumorigenicity independent of EGFR signaling. HELDR globally binds genomic DNA and recruits the transcription co-activator p300 to the KAT7 promoter. p300-induced H3K27ac at the KAT7 promoter enlists other co-transcription factors, activating KAT7 transcription. KAT7 induces H3K14ac and H4K12ac that activate KAT7-driven gene programs that are critical for GBM malignancy. Targeting KAT7 or HELDR markedly enhances therapeutic effects of anti-EGFR treatments for GBM. These results not only reveal the role of HELDR in EGFR-driven GBM but also provide a strong rationale to characterize the role of lncRNAs co-amplified with driver oncogenes in human cancers.

## Introduction

Glioblastoma (GBM) is a commonly diagnosed and the most malignant adult primary brain tumor^1^. Despite treatment consisting of maximal surgical resection followed by radiation therapy (RT) and chemotherapy, along with extensive testing of targeted- or immuno- therapies, most patients with GBM will only live 14 to 16 months^2,3^. Among GBM that is isocitrate dehydrogenase (IDH) wild type under the new 2021 WHO classification^1^, > 55% tumors have *EGFR* amplification and/or mutations^4^. EGFR is an oncogenic driver of GBM that is frequently amplified within extrachromosomal DNAs (ecDNAs)^5,6^. ecDNAs are megabase-sized, double-stranded circular DNAs in cancer cell nuclei that drive oncogene amplification, dysregulated gene expression and intratumoral heterogeneity, thereby promoting cancer malignancy^7^. Due to its oncogenic driving role, EGFR has been designated as a biomarker and therapeutic target for GBM treatments. However, EGFR-targeting therapies remain clinically ineffective for GBM^8^. The failures of targeting EGFR are largely attributed to GBM heterogeneity with intertwined oncogenic signaling, dynamic switching between tumor subtypes, and an immuno-suppressive tumor microenvironment^9^. While the canonical downstream signaling of EGFR is well studied, other non-canonical pathways remain to be discovered.

Histone lysine acetylation catalyzed by the lysine acetyltransferase (KAT) family epigenetically regulates gene expression by regulating chromatin configuration^10^. The KAT family includes abundant subtypes of enzymes that selectively catalyze different types of histones or non-histone acetylation^10^. p300 (also known as KAT3B) mediates histone H3 lysine 27 acetylation (H3K27ac), a marker of active promoters and enhancers^11^, and functions as a transcriptional co-activator^12^. p300 does not directly bind DNA, instead p300 is recruited by other DNA-binding factors^13^ or long non-coding RNAs (lncRNAs)^14^. KAT7 (also known as HBO1 or MYST2), is a member of the KAT family that primarily catalyzes H3 and H4 acetylation^15,16^. KAT7 is a multifunctional protein critically involved in cellular functions including DNA replication, development, cell proliferation, genome stability, DNA repair, senescence, and cancer^15,16^. KAT7 inhibition shows anti-tumor effects in several human malignancies^17^, however, its role in GBM remains under studied.

LncRNAs are defined as transcripts that are longer than 200 nucleotides and do not appear to encode proteins. LncRNAs have cell-, tissue-, and tumor-specific expression and functions, and play critical roles in normal development as well as disease processes, including those associated with cancer^18^. LncRNAs modulate cellular functions through RNA-RNA, RNA-DNA and RNA-protein interactions, with their emerging roles in controlling transcriptions^19,20^. LncRNAs can activate or suppress cellular signaling pathways, thereby affecting tumor malignancy as well as tumor response to therapies^21^. LncRNA expression profiles and the tumor-modulating functions have been studied in cancers, including GBM^22,23^. Recent studies revealed that a large number of lncRNAs, either annotated or unannotated, are co-amplified with oncogene drivers at ecDNAs, such as *MYC* and *MDM2,* in human cancers including GBM^24,25^. However, the roles of lncRNAs associated with ecDNAs in EGFR-driven tumorigenesis and GBM therapy resistance remains unanswered.

In this study, we identified a de novo gene that is predominantly transcribed from the antisense strand of the EGFR long non-coding downstream RNA (*ELDR*) located near *EGFR* within ecDNAs in GBM^5,6,26^. This novel gene is co-amplified with *EGFR*in GBM. We named it hidden *ELDR(HELDR). HELDR* binds to promoters, gene bodies, and intergenic regions, regulating global gene expression without affecting EGFR signaling. Mechanistically, *HELDR* binds to and recruits p300 to the promoter of *KAT7,* resulting in increased levels of H3K27ac, recruitment of transcription factors, and enhanced transcription of KAT7. Last, the *HELDR*-KAT7 axis facilitates the expression of genes associated with GBM resistance to EGFR inhibitors (EGFRi) and targeting KAT7 or *HELDR* significantly enhances GBM responses to EGFRi treatments in vivo.

## Results

### LncRNA ELDR is co-amplified with EGFR in GBM

**Figure 1:**
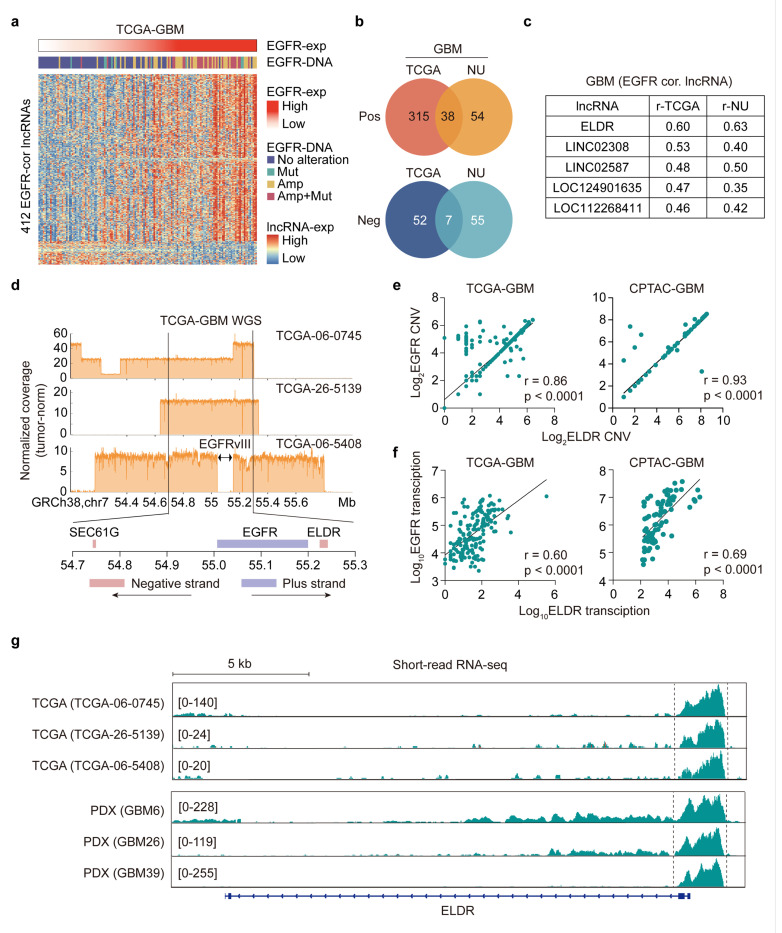
The IncRNA *ELDR* is co-amplified with *EGFR* in GBM. **a,** Heatmap depicts expressions of 412 IncRNAs that exhibited significant correlation with *EGFR* expression in TCGA-GBM samples (Spearman method, |r| > 0.3 and p < 0.05). Expression levels of IncRNAs and EGFR, and status of *EGFR* gene amplification and mutation are indicted. **b,** Venn diagrams for common lncRNAs that significantly correlate with *EGFR* expression in TCGA and NU GBM samples (Spearman method, |r| > 0.3 and p < 0.05). **c,** Top 5 lncRNAs that positively correlated with EGFR expression. **d,** WGS data of three representative TCGA GBM samples for *EGFR* or *EGFRvIII* gene copy numbers. *EGFR* or *EGFRvIII and ELDR* gene loci are co-amplified. **e,** The correlation of CNV between *EGFR* and *ELDR* gene loci in TCGA and CPTAC GBM samples (Spearman method). **f,** The correlation in RNA transcripts between *EGFR* and *ELDR* in TCGA and CPTAC GBM samples (Spearman method). **g,** The RNA-seq data (IGV) of three representative TCGA samples (same cases in **d**) and three Mayo clinic PDX GBM samples. Abbreviation: Mut, mutation; Amp, amplification; NU, Northwestern University; WGS, whole genome sequencing; CNV, copy number variation; TCGA, The Cancer Genome Atlas; CPTAC, Clinical Proteomic Tumor Analysis Consortium; PDX, Patient-Derived Xenografts; IGV, Integrative Genomics Viewer.

To study the involvement of lncRNAs in EGFR-driven GBM malignancy, we performed Pearson correlation analyses using RNA-seq data from The Cancer Genome Atlas (TCGA) and our Northwestern University (NU) cohort^27^ (Supplementary Tables 1 and 2). We identified a total of 412 and 154 lncRNAs whose expressions correlate with EGFR expression in TCGA and NU datasets, respectively (Fig. 1a, Extended data Fig. 1 a). Among these, 38 are positively correlated and 7 lncRNAs are negatively correlated with EGFR expression on both datasets (Fig. 1b) where *ELDR* was the top hit (Fig. 1c). *ELDR* is an annotated lncRNA^26^ transcribed from 7p11, located near *EGFR* within ecDNAs in GBM^5,6^. Analysis of whole genome sequencing data of three TCGA GBM tumors revealed that *ELDR* is situated within the *EGFR* amplicon or within ecDNAs that also contains several other co-amplified genes (Fig. 1d)^5,28^. *ELDR* expression is significantly upregulated in GBM in relation to normal brain and low-grade gliomas (LGG, Extended data Fig. 1 b) and is positively correlated with *EGFR* gene copy number (Fig. 1 e) and RNA transcript levels (Fig. 1f) in GBM. *ELDR* transcription peaks in three TCGA GBM tumors are also found in patient-derived xenograft (PDX) models that have *EGFR* amplification^29^ (Fig. 1g). Moreover, while *ELDR* expression peaks aligned perfectly between TCGA GBM and PDX tumors, the major expression peaks are outside the first exon of the *ELDR* gene (Fig. 1g). Last, expression of *ELDR* correlates with overall survival of GBM and is markedly elevated in GBM compared to other cancers in TCGA datasets (Extended data Fig. 1c–d).

### A de novo lncRNA, HELDR is the major transcript at the ELDR locus

**Figure 2:**
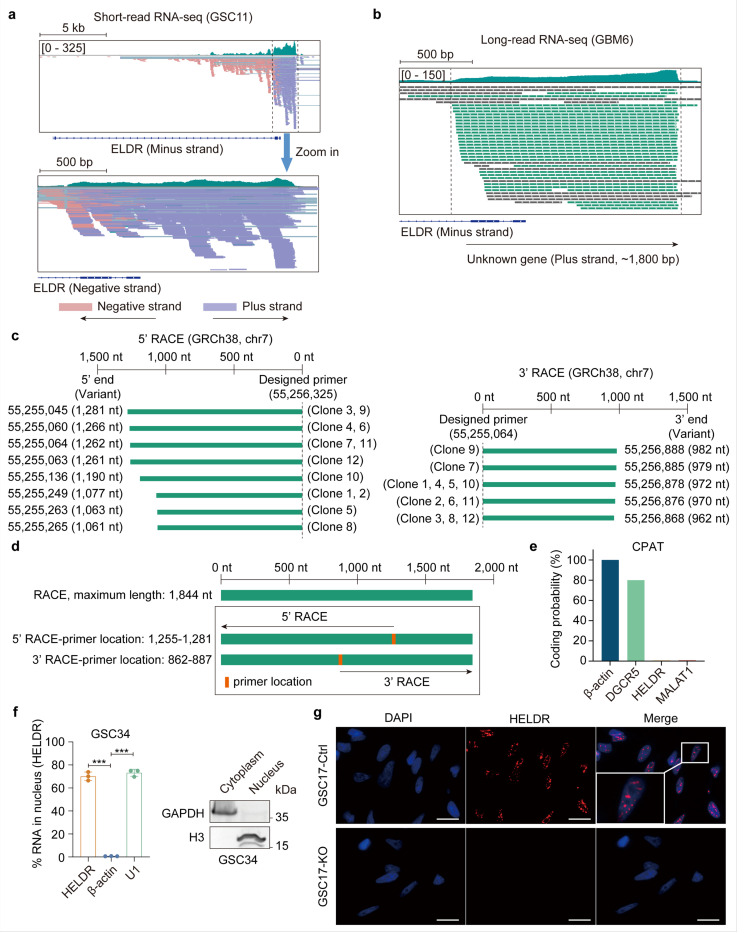
De novo IncRNA *HELDR* is the major transcript in the *ELDR* locus and localized in the nucleus. **a,** Shortread RNA-seq of GSC11 in the *ELDR* locus is shown in IGV. Light pink and lavender reads represent RNA matching indicated strands. **b,** Long-read RNA-seq of PDX GBM6 in the *ELDR* locus is shown in IGV. Green reads represent RNA transcribed from the plus strand (unknown gene). **c,** Data of 5’ RACE and 3’ RACE for *HELDR in* GBM6 cells. Twelve clones of each were collected for Sanger sequencing. **d,** The maximum length obtained in the RACE and location of primers are shown. **e,** An online software CPAT (https://wlcb.oit.uci.edu/cpat/) was utilized to assess the coding potential of the newly identified *HELDR.* Genes of *b-actin, DGCR5,* and *MALAT1* are controls. **f,** Left, qRT-PCR for gene expression in the nucleus. *b-actin* and *U1* are controls. Right, IB for cellular fraction markers. GADPH is a cytosolic protein control and H3 is a nucleus protein control. **g,** Single-molecule RNA fluorescence in situ hybridization (smFISH) for *HELDR* localization in GSC17 cells. *HELDR*knockout (KO) cells are used as negative control. Abbreviation: Ctrl, control. Scale bar, 20 μm. ***p< 0.001, by one-way ANOVA. Data are presented as mean ± s.d. in (**f**).

Public RNA-seq datasets including TCGA, usually provide levels of RNA transcripts without information about the transcribing strand^30^. Lack of the transcribing strand information that the RNA-seq reads were originated results in inaccurate assessment of gene profiling and missing genes that could be important, especially when gene locus overlaps. We further analyzed *ELDR* transcripts in a RNA seq dataset^5^ and two of our in-house glioma stem-like cell (GSC) RNA-seq datasets with strand direction information and found that RNA transcribed from the *ELDR* locus primarily comes from the plus strand, while the annotated *ELDR* is transcribed from the negative strand with much less abundance compared to the transcripts originated from the plus strand (Fig. 2a, Extended data, Fig. 2a, b). This data indicates that a previously unidentified transcript could be the major gene product at the *ELDR* locus but is transcribed from the antisense strand in relation to *ELDR.* Long-read RNA-seq on patient-derived xenograft (PDX) GBM6 cells with *EGFR* amplification^29^ revealed that a group of RNA transcripts with only one exon and slightly varying lengths was the main transcript of this locus (Fig. 2b). Analyses of three short-read RNA-seq datasets validated this observation (Fig. 2a, Extended data Fig. 2a, b). Furthermore, rapid amplification of cDNA ends (RACE) analyses confirmed the length and the sequence of this unannotated gene (Fig. 2c, d, Extended data Fig. 2c–e, Supplementary Table 3).

Since this new gene transcript lacks introns and exhibits various lengths, we reasoned that it could be a non-coding RNA. Using the online software ORF Finder^31^ and two orthologous algorithms (CPAT and CPC)^32,33^ revealed no open reading frames (ORFs) longer than 300 nucleotides in this transcript (Fig. 2e, Extended data Fig. 2e, f), suggesting that it lacks the capacity to encoding a protein. Additionally, this transcript is primarily localized in the cell nucleus (Fig. 2f, g, Extended data Fig. 2g). Taken together, we identified a de novo lncRNA that is transcribed from the plus strand at the *ELDR* gene locus and is currently unannotated. We named it hidden *ELDR (HELDR).*

### HELDR is critical for the tumorigenicity of GBM with EGFR-HELDR co-amplification

**Figure 3:**
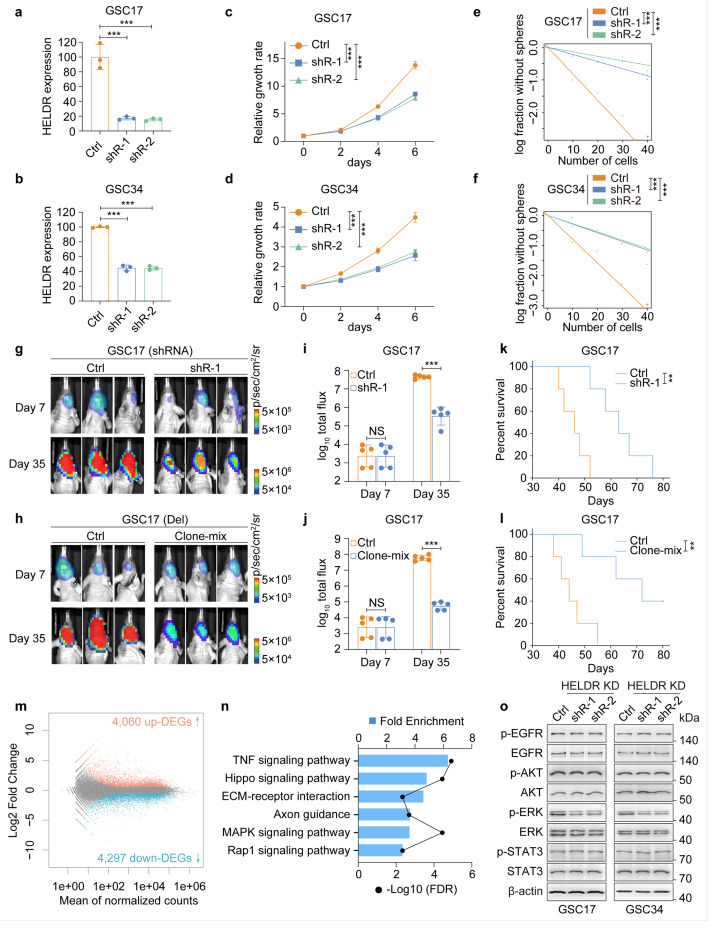
*HELDR* is critical for cell proliferation and tumorigenicity and globally regulates gene expression independent of EGFR in GSCs with *EGFR* amplification. **a, b,** qRT-PCR for shRNA knockdown (KD) of *HELDR* in GSC17 and GSC34 cells that *EGFR* is amplified. **c, d,** Cell proliferation for GSC17 and GSC34 cells with or without *HELDR* KD. **e, f,** Glioma sphere formation (self-renewal capacity) of GSC17 and GSC34 cells with or without *HELDR* KD. **g, h,** Representative bioluminescence imaging (BLI) images of indicated GSC17 tumor xenograft-bearing mice. Control groups were compared with *HELDR* KD or mixed clones of CRISPR-Cas9-mediated *HELDR* deletion (Del) (Extended data Fig. 3) at day 7 and day 35 post-implantation. **i, j,** Quantification of BLI signals. **k, l,** Kaplan-Meier analysis of GSC17 tumor xenograft-bearing mice. **m,** A MA Bland-Altman plot displaying the log_2_FC and mean of normalized counts for each gene of RNA-seq data of GSC17 cells with *HELDR* KD versus the control. Two biological replicates were analyzed. Red indicates upregulated differentially expressed genes (DEGs) (fold change, FC > 1.5, padj < 0.05), while blue indicates downregulated DEGs (FC < −1.5, padj < 0.05). **n,** KEGG gene enrichment analysis for significantly downregulated DEGs in GSC17 with *HELDR* KD versus the control (FC < −4, padj < 0.05). **o,** I B for EGFR and EGFR downstream signaling proteins in GSC17 or GSC34 cells with or without *HELDR* KD. NS, not significant; **p < 0.01, ***p < 0.001, one-way ANOVA in **a-d,** likelihood ratio test in **e** and **f**, unpaired Student’s *t*-tests in **i** and **j**, log-rank test in **k** and **l**. Data are presented as mean ± s.d. (**a-d, i** and **j**).

To study the potential role of *HELDR* in *EGFR*-driven GBM tumorigenesis, we determined the expression levels of *HELDR* in GSCs and patient-derived GBM PDX lines with known *EGFR* expression^29,34,35^. As expected, expression levels of *HELDR* are positively correlated with that of EGFR in these GBM models (Extended data, Fig. 3a). We next knocked down endogenous *HELDR* in GSCs with *EGFR* amplification^34^ using two separate shRNAs (Fig. 3a, b). Knockdown (KD) of HELDR markedly impeded proliferation and self-renewal ability of the GSCs in vitro (Fig. 3c–f). In addition, we also used CRISPR/Cas9-mediated knockout (KO) to deplete *HELDR* in GSC17 cells (Extended data Fig. 3b–e). Consistently, *HELDR* KO also significantly impaired GSC17 tumorigenic properties in vitro (Extended data Fig. 3f, g). In vivo, both *HELDR* KD and depletion markedly suppressed the growth of orthotopic GSC17 tumor xenografts and improved the survival of animals bearing GSC brain tumor xenografts (Fig. 3g–l). Taken together, this data indicates that *HELDR* supports *EGFR*-driven GBM tumorigenicity.

### HELDR regulates global gene expression but does not affect EGFR expression or its downstream signaling

To investigate how *HELDR* regulates *EGFR*-driven GBM tumorigenesis, we performed RNA-seq analysis on GSC17 cells where endogenous *HELDR* was knocked down and identified more than 8,000 differentially expressed genes (DEGs) in the modified GSCs (Fig. 3m, Supplementary Table 4). Since *HELDR* promotes EGFR-driven GBM tumorigenicity, we focused on the down-regulated DEGs. Kyoto Encyclopedia of Genes and Genomes (KEGG) analysis reveals several enriched pathways including TNF-, MAPK-, and Hippo-pathways (Fig. 3n) that are known to promote GBM tumorigenesis and resistance to EGFRi therapy^9,36–38^.

LncRNAs, particularly those that are bi-directionally transcribed, can act as enhancer RNAs and function in cis or trans to regulate the transcription of adjacent genes^20^. Thus, we examined the expressions of coding genes located near the *ELDR/HELDR* locus in GSCs (Extended data Fig. 4a). Most genes, including EGFR, showed no significant changes in expression levels in GSC17 with or without HELDR KD (Extended data Fig. 4b). We also confirmed that EGFR signaling remains unaltered after HELDR KD (Fig. 3o). Thus, *HELDR* that is co-amplified with *EGFR,* globally regulates gene expression but does not influence EGFR expression or its downstream signaling in GBM.

### HELDR globally binds genomic DNA

**Figure 4:**
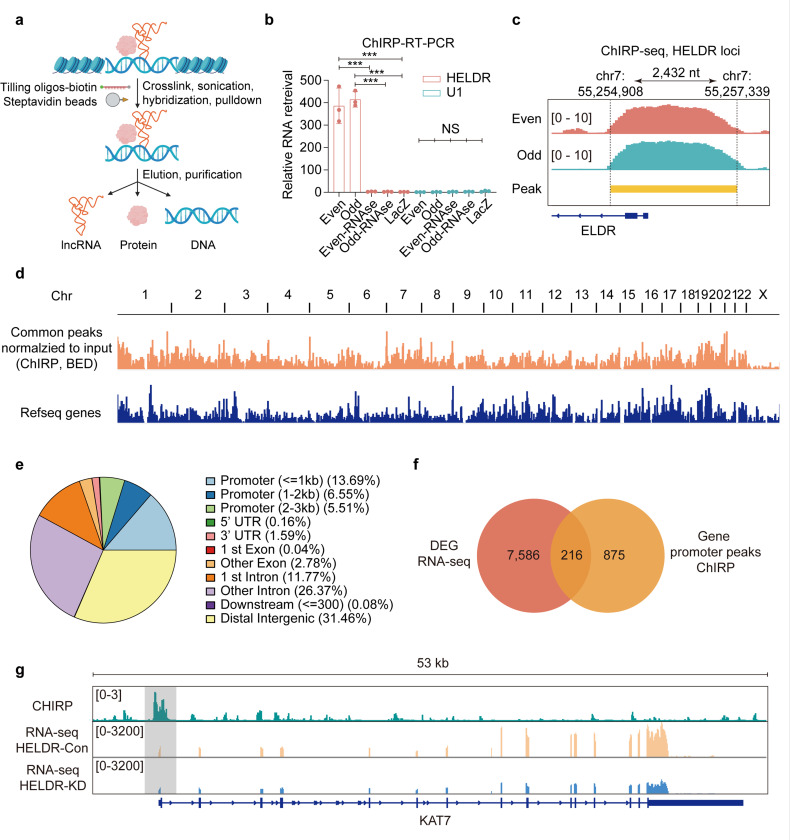
*HELDR* globally binds to genomic DNA. **a,** Schematic diagram of Chromatin Isolation by RNA Purification (ChIRP) analysis. **b,** qRT-PCR for RNA levels of *HELDR* and the negative control U1 retrieved from ChIRP. Even and odd, probe pools with even-numbered or odd-numbered probes targeting the *HELDR* locus sequentially. LacZ, probe pools targeting Escherichia coli-expressed lacZ as a negative control. **c,** ChIRP-seq shown in IGV in the *HELDR* locus. **d,** Common peaks between even and odd groups (Browser Extensible Data, BED file) in *HELDR*ChIRP-seq analysis across the whole genome. **e,** Pie chart shows the distribution of *HELDRpeaks* across indicated genomic features. **f,** The Venn diagram for the subgroup of genes with DEGs in RNA-seq and common ChIRP peak in the promoter region. **g,** IGV views of *KAT7*in *HELDR* ChIRP-seq and RNA-seq. The peaks of HELDR ChIRP-seq in the promoter region are highlighted. NS, not significant; ***p < 0.001, by one-way ANOVA. Data are presented as mean ± s.d. in (**b**).

One common mechanism by which lncRNAs globally regulate gene expression is to bind genomic DNA and fine-tune gene transcription by regulating the formation of transcription complexes, epigenetic modifications, or 3D genome architecture^19,20^. Thus, we employed Chromatin Isolation by RNA Purification (ChIRP)^39,40^ using two independent sets of probes to identify the genomic DNA binding sites of endogenous *HELDR* (Fig. 4a). *HELDR* shows specific RNA and DNA enrichment in both the even and odd probe groups, but not in negative control groups (Fig. 4b, Extended data Fig. 4c). In addition, specific ChIRP-seq peaks were observed in both the even and odd groups at the DNA locus from which *HELDR* is transcribed (Fig. 4c). This data further confirms the presence of *HELDR* at its gene locus and underscores the specificity of the ChIRP assay.

A total of 7,459 common peaks between the even and odd groups were identified in the ChIRP-seq experiment (Supplementary Table 5). These peaks are distributed across the entire genome (Fig. 4d), suggesting a global and significant regulatory role for *HELDR.* We observed that approximately a quarter of the peaks are localized in promoter regions (Fig. 4e), which are crucial for transcription regulation. Therefore, we focused on the overlapping genes between the DEGs identified in the RNA-seq analysis and those with common peaks in their promoters in the ChIRP-seq data. Ultimately, we identified 216 genes that could be directly regulated by *HELDR* via transcription (Fig. 4f, Supplementary Table 6).

### HELDR recruits p300 to the KAT7 promoter and enhances KAT7 transcription through an epigenetic mechanism

**Figure 5:**
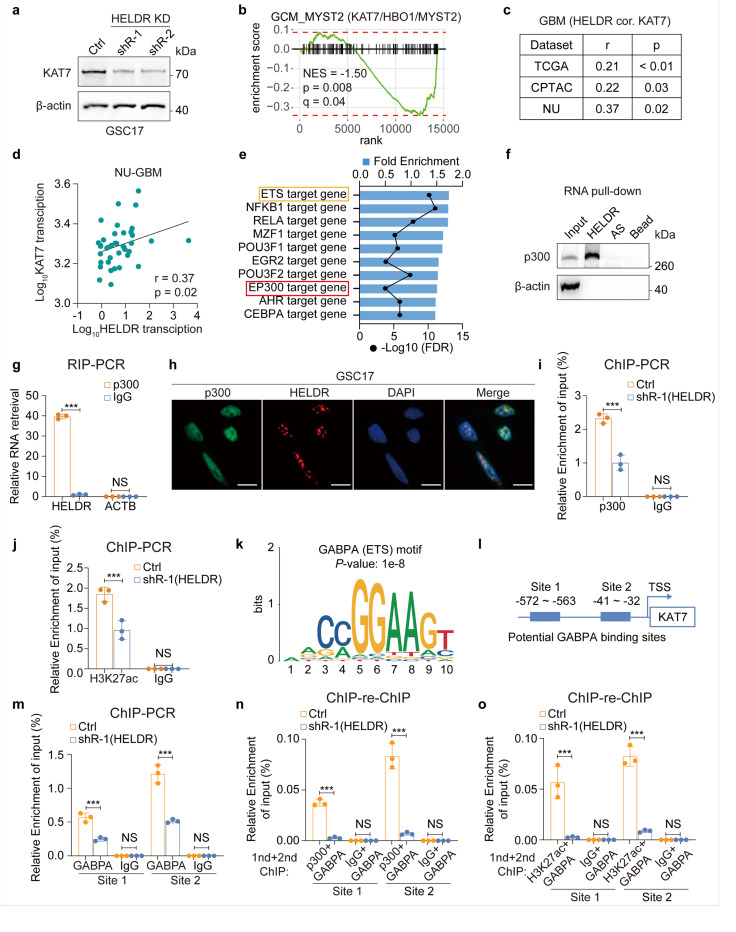
*HELDR* epigenetically regulates *KAT7* expression. **a,** IB for KAT7 proteins in GSC17 cells with or without *HELDR* KD. **b,** Gene Set Enrichment Analysis (GSEA) analysis of significantly DEGs (|FC| < 2, padj < 0.05) induced by *HELDR* KD in GSC17 cells. **c, d,** Spearman analysis of RNA-seq data for *HELDR* RNA in relation to *KAT7*in GBM of indicated datasets. **e,** Gene enrichment analysis using the TF.TARGET.REGNETWORK gene set shows significantly downregulated DEGs (FC < −2, padj < 0.05) after *HELDRKD* in GSCs. **f,** RNA pull-down. In vitro transcribed *HELDR* binds to p300 in GSC17 cell extract. Antisense (AS) transcript, b-actin, and beads are controls. **g,** RNA immunoprecipitation (RIP). Endogenous *HELDR* binds to p300 proteins in GSC17 cells. IgG and b-actin (ACTB) transcript are controls. **h,** Images of smFISH for *HELDR* and immunofluorescence (IF) staining for p300 in GSC17 cells. **i, j,** Chromatin immunoprecipitation (ChIP)-qPCR. Enrichment of p300 or H3K27ac at KAT7 gene promoters were compared in GSC17 cells with or without *HELDR* KD. **k,** Sketches for sequence logo plot of GABPA and enrichment in *HELDR* ChIRP-seq. **I,** Predicted GABPA binding site in the promoter of *KAT7* using an online software: Jaspar. **m,**ChIP-qPCR. Enrichment of GABPA at *KAT7* gene promoters were compared in GSC17 cells with or without *HELDR* KD. **n, o,** Sequential chromatin immunoprecipitation (ChIP-re-ChIP-qPCR). Enrichment of P300/GABPA or H3K27ac/GABPA complexes at *KAT7* gene promoters were compared in GSC17 cells with or without *HELDR* KD. The first ChIP was performed with p300, H3K27ac, or IgG, followed by a second ChIP using GABPA. In (**i-j**) and (**m-o**), IgG is used as a control. Scale bar, 10 μm. NS, not significant; ***p< 0.001, by unpaired Student’s *t*-tests. Data are presented as mean ± s.d. (**g, i, j** and **m-o**).

Among the 216 candidates, we present the localization patterns of several representative genes, including UABSH3B, DEPDC1, and KAT7 (Fig. 4g, Extended data Fig. 4d). Lysine acetyltransferase 7 (KAT7, also known as HBO1 or MYST2) drew our attention. KAT7 has been shown to play an important role in human cancers^41–43^, but its role in GBM is largely unknown. Our analysis indicated that *HELDR* binds to the promoter of KAT7, and *HELDR* KD significantly decreased KAT7 expressions at both the RNA level and decreased protein levels (Fig. 4g, 5a, Extended data Fig. 5a). Gene Set Enrichment Analysis (GSEA) revealed significant enrichment changes of KAT7 (MYST2)-related genes following HELDR KD (Fig. 5b, Extended data, Fig. 5b). Additionally, a positive correlation was found between *HELDR* and *KAT7* in TCGA, CPTAC, and NU GBM datasets (Fig. 5c, d, Extended data Fig. 5c). These data suggest that *HELDR* plays a role in regulating the expression of KAT7.

We hypothesized that the binding of *HELDR* at gene promoters could either facilitate or impede the binding of transcription-related proteins to these regions, thereby regulating transcription. Thus, we conducted gene enrichment analysis on our RNA-seq data and identified potential transcription factors or epigenetic regulators associated with *HELDR* (Fig. 5e). Among the top candidates, we focused on p300 since p300 is an epigenetic regulator, and two other lncRNAs bind to and recruit p300 to their targeting gene enhancers^14,44^, whereas the most remaining candidates are general transcription factors that lack well-defined RNA-binding domains^45^. We found that *HELDR* binds to p300 (Fig. 5f, g) and co-localizes with p300 in the nucleus (Fig. 5h, Extended data Fig. 5d).

p300 induces H3K27ac at targeted gene promoters, enhancers and super enhancers, and recruits transcription factors to activate transcription^14,44,46^. As anticipated, *HELDR* plays a role in the enrichment of p300 and H3K27ac at the *KAT7* promoter (Fig. 5i, j, Extended data Fig. 5e). Further, motif analysis of the ChIRP-seq data revealed that *HELDR* tends to bind to motifs of several members of the erythroblast transformation-specific (ETS) transcription factor family (Fig. 5k, Extended data Fig. 5f), consistent to the enrichment of ETS-targeted genes in *HELDR*-regulated DEGs (Fig. 5e). We selected GA-binding protein, alpha subunit (GABPA) as a representative target due to its established role in GBM^47,48^. *HELDR* KD markedly reduced GABPA binding at the *KAT7* promoter but had no effect on GABPA’s protein expression (Fig. 5m, Extended data Fig. 5g, h). Furthermore, HELDR KD also significantly hindered the enrichment of transcription complexes (p300/GABPA or H3K27ac/GABPA) at the *KAT7* promoter as demonstrated by sequential chromatin immunoprecipitation (ChlP-re-ChIP) (Fig. 5n, o, Extended data Fig. 5i, j).

### KAT7 mediates HELDR-regulated GBM tumorigenic properties

**Figure 6:**
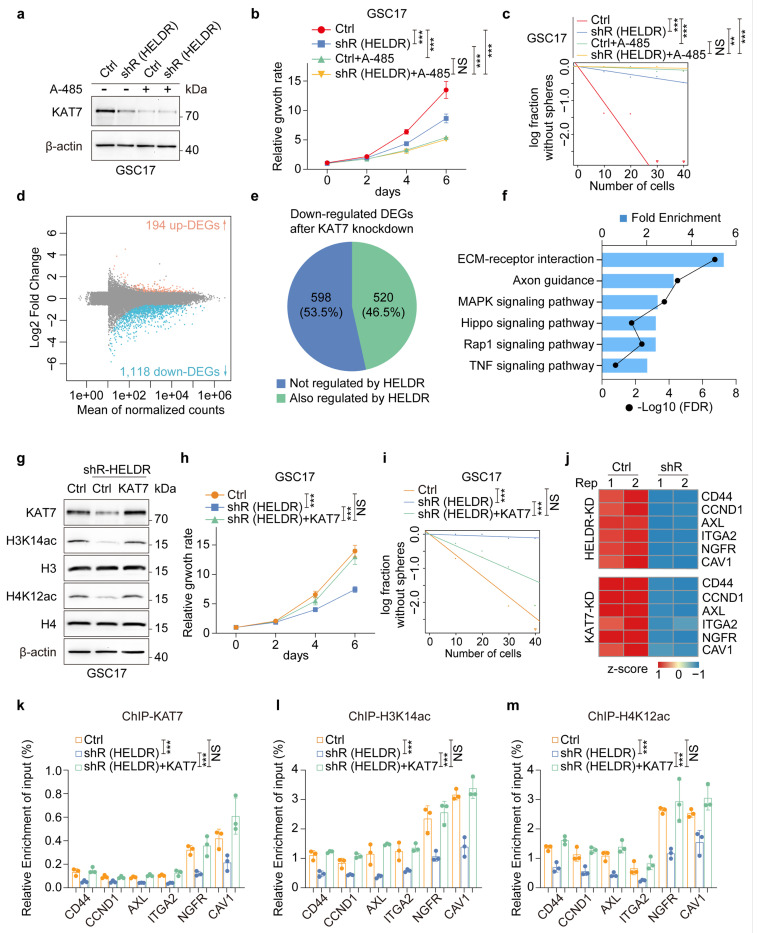
KAT7 mediates *HELDR*-promoted GBM tumorigenesis. **a,** IB for KAT7 protein expression in GSC17 cells with indicated modification and treatment (5 μM A-485) for three days. **b, c,** cell proliferation (**b**), and glioma sphere formation (**c**) of GSC17 cells with indicated modification and treatment. GSC17 cells were treated with 5 μM A-485. **d,** MA Bland-Altman plot displaying the log_2_FC and mean of normalized counts for each gene of RNA-seq data in GSC17 KAT7 KD or control cells with two biological replicates. Light pink dots, upregulated DEGs (FC > 1.5, padj < 0.05), blue dots, downregulated DEGs (FC < −1.5, padj < 0.05). **e,** Pie graph. KAT7 KD down-regulated DEGs that are not regulated by *HELDR* KD (KAT7-specific, 53.3%) or also regulated by *HELDR* KD (46.5%) in GSC17 cells. **f,** KEGG analysis of significantly down-regulated DEGs in GSC17 KAT7 KD cells. **g-i**, Overexpression of KAT7 rescued KAT7 KD-suppressed H3K14ac and H412Kac (IB in **g**), cell proliferation (**h**), and glioma sphere formation (**i**) in GSC17 cells. **j,** Common genes that regulated by both KAT7 KD and *HELDR* KD in GSC17 cells with two biological replicates. **k-m,** ChlP-qPCR. Enrichment of KAT7 (**k**), H3K14ac (**l**), and H4K12AC (**m**) at the promoters of indicated genes in GSC17 cells, with or without *HELDR* KD or rescue by KAT7 overexpression. NS, not significant; **p < 0.01, ***p < 0.001, one-way ANOVA in **b, h** and **k-m**. likelihood ratio test in **c** and **i**. Data are presented as mean ± s.d. (**b, h** and **k-m**).

To demonstrate that p300 is essential for *HELDRs* function and the underlying mechanism, we treated GSCs with a selective p300 inhibitor, A-485, that inhibits p300-induced histone acetylation^49^. Like the inhibitory effects of *HELDR* KD, A-485 treatment decreased KAT7 protein expression and attenuated cell proliferation and self-renewal capacity of GSCs (Fig. 6a–c, Extended data Fig. 6a–c). These results indicate that *HELDR* binds to the promoter of *KAT7,* facilitating the formation of the transcription complex and activating p300-mediated gene transcription.

Next, we assessed the effects of *HELDR* binding to the *KAT7* promotor on GBM tumorigenesis. KAT7 induces H3 and H4 acetylation, primarily H3K14 and H4K12^15^. Indeed, KAT7 KD markedly reduced H3K14ac and H4K12ac (Extended data Fig. 6d), and decreased proliferation and self-renewal capacity of GSCs (Extended data Fig. 6e–h). This data is consistent with other studies showing that KAT7-induced histone acetylation activates gene transcription and that KAT7 KD decreases proliferation^50,51^. KAT7 KD resulted in significantly more down-regulated DEGs than up-regulated DEGs in modified GSCs (Fig. 6d, Supplementary Table 7). Interestingly, among the 1,118 of down-regulated DEGs induced by KAT7 KD, 520 genes were also regulated by *HELDR* (Fig. 6e, Supplementary Table 8). Pathway analysis revealed that the top enriched pathway caused by *HELDR* KD shown in Fig. 3n is also enriched in KAT7 KD GSCs (Fig. 6f). Furthermore, re-expression of KAT7 in *HELDR-KD* GSCs restored the reduced levels of H3K14ac and H4K12ac, as well as rescued the impaired proliferation and self-renewal capacity caused by *HELDR* KD in GSCs (Fig. 6g–i, Extended data Fig. 6i–k).

KAT7 activates gene transcription by inducing histone acetylation at gene promoters^50,52^. Thus, we investigated whether *HELDR* and KAT7 regulate histone acetylation at the promoters of key downstream genes in the *HELDR-KAT7* axis. We focused on several representative genes with important functions in GBM such as *CD44,* cyclin D1 *(CCND1*), AXL receptor tyrosine kinase (*AXL*), integrin subunit alpha 2 (*ITGA2*), nerve growth factor receptor *(NGFR),* and caveolin 1 *(CAV1*)^36,53–56^. The transcription of these genes is down-regulated in GSCs following KD of *HELDR* or KAT7 (Fig. 6j, Supplementary Table 4 and Supplementary Table 7). As expected, KD of *HELDR* or KAT7 markedly decreased while KAT7 overexpression rescued the enrichment of KAT7, H3K14ac, and H4K12ac at the promoters of these genes in GSCs (Fig. 6k–m, Extended data Fig. 6l–o), Together, these results indicate that the KAT7-H3K14ac/H4K12ac axis is a critical downstream mediator for *HELDR*-regulated GBM tumorigenic properties.

### Targeting KAT7 enhances the effects of anti-EGFR therapy in preclinical GBM models

**Figure 7:**
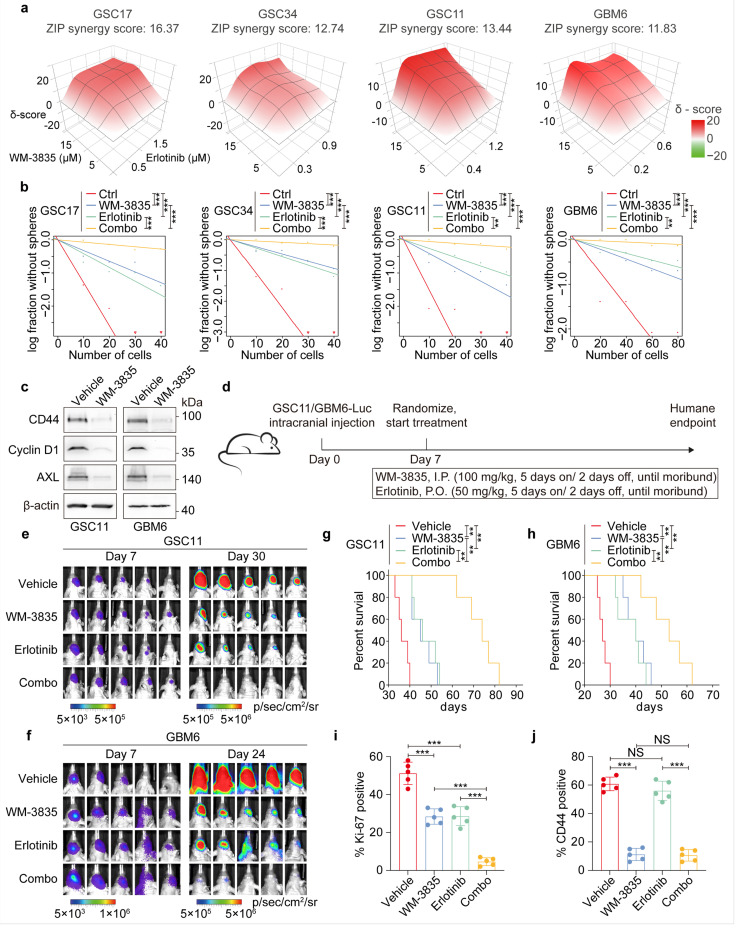
KAT7 inhibitor WM-3835 synergistically enhanced anti-GBM activity of EGFR inhibitor Erlotinib. **a,** ZIP synergy scores calculated by SynergyFinder (https://synergyfinder.fimm.fi/) for EGFR-amplified GSCs (GSC17, GSC34, GSC11) and GBM6 that were treated with WM-3835) and erlotinib for 6 days with indicated concentrations. Synergy scores were assessed based on the effects on GSC cell proliferation. **b,** Glioma sphere formation of indicated GSCs that were treated separately or in combination with indicated inhibitors. GSC17: WM-3835, 10 μM; erlotinib, 1 μM; GSC34: WM-3835, 10 μM; erlotinib, 0.6 μM; GSC11: WM-3835, 10 μM; erlotinib, 0.8 μM; GBM6: WM-3835, 10 μM; erlotinib, 0.4 μM. **c,** IB for indicated protein expressions in GSC11 or GBM6 cells treated with vehicle or WM-3835 for 72 h. **d,** The workflow of the animal experiment. **e, f,** BLI images, **g, h,** Kaplan-Meier analysis for mice bearing indicated GSC or GBM brain tumor xenografts with indicated treatments. **i, j,** Quantification of IF staining (Extended data Fig. 7) of brain sections with GBM6 tumor xenografts with indicated treatments. **p< 0.01, ***p < 0.001, likelihood ratio test in **b,** log-rank test in **g** and **h**, one-way ANOVA in **i** and **j**. Data are presented as mean ± s.d. (**i** and **j**).

Next, we tested the effects of targeting KAT7 using a potent acetyl-CoA competitive inhibitor against KAT7, WM-3835^17^ with anti-EGFR therapy for GBM. In vitro, the KAT7 inhibitor WM-3835 exhibited synergistic effects when combined with the EGFR inhibitor Erlotinib in suppressing cell proliferation and self-renewal capacity of multiple GSCs or patient-derived GBM6 cells that *EGFR* is amplified (Fig. 7a, b, Extended data Fig. 7a). Then, we assessed the effect of KAT7 inhibition by WM-3835 on *HEDLR*/KAT7-regulated genes critical for GBM tumorigenesis and resistance to anti-EGFR therapy^9,36–38^(Fig. 6j). As expected, WM-3835 treatment decreased protein abundance of CD44, cyclin D1 *(CCND1*), AXL receptor tyrosine kinase *(AXL),* integrin subunit alpha 2 *(ITGA2),* nerve growth factor receptor *(NGFR),* and caveolin 1 *(CAV1*) (Fig. 7c, Extended data Fig. 7b).

Last, we evaluated the therapeutic effects of individual or combination therapy with the KAT7 inhibitor WM-3835 and the EGFR inhibitor Erlotinib on orthotopic GSC11 and GBM6 tumor xenograft models (Fig. 7d). Both WM-3835 and Erlotinib monotherapy showed growth suppressive effect on intracranial tumors, as indicated by bioluminescence monitoring of the tumor response to treatment as well as by survival analyses. Importantly, combination treatments showed significantly increased anti-tumor activity, relative to monotherapy, with co-administration with WM-3835 and Erlotinib increasing median overall survival from 45 and 46 days to 74 days in the GSC11 xenograft model and from 40 and 40 days to 53 days in GBM6 (Fig. 7e–h). Intracranial tumors were examined for treatment effect on proliferation, apoptosis, and molecular targets of the KAT7 inhibitor (CD44, H3K14ac, H4K12ac), and the EGFR inhibitor (p-EGFR). We found that GSC11 and GBM6 tumor xenografts treated with combined WM-3835 + Erlotinib had significantly lower proliferation indices (by Ki-67 staining) and higher apoptosis levels (by cleaved caspase 3) when compared to WM-3835 or Erlotinib treatment (Fig. 7i, j, Extended data Fig. 7c, d). This data shows that inhibition of KAT7 enhanced cytotoxicity of EGFRi against GBM tumor xenografts in vivo.

### Antisense oligonucleotide (ASO) targeting of HELDR enhances the effects of anti-EGFR therapy for GBM

**Figure 8:**
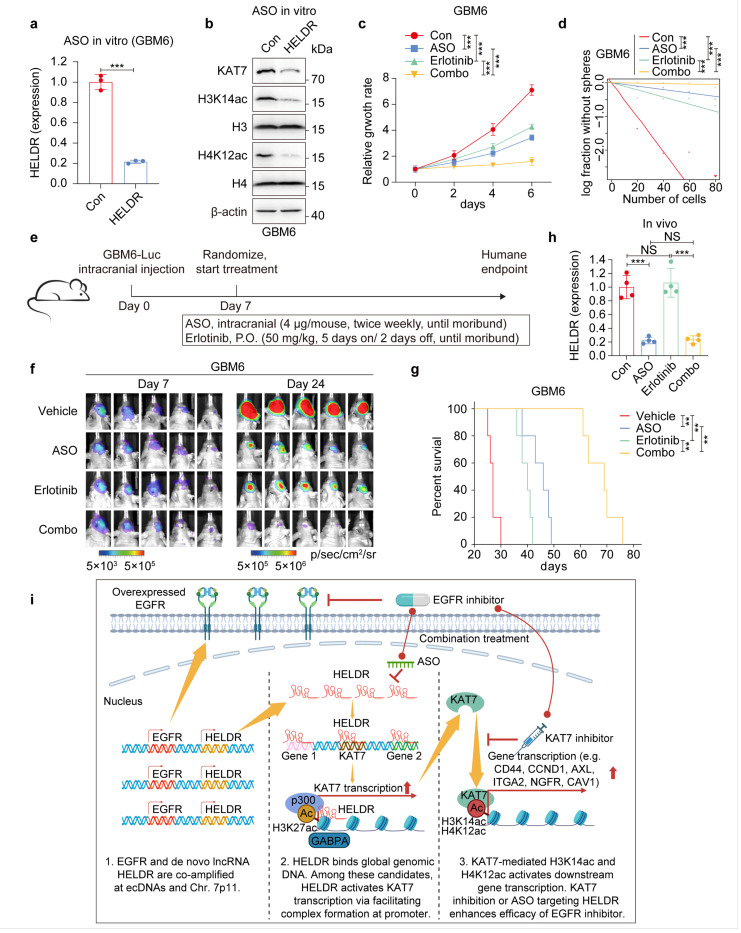
ASO-targeting *HELDR* synergistically enhanced anti-GBM activity of EGFR inhibitor Erlotinib. **a,** qRT-PCR analysis of GBM6 cells 24 hours after transfection with 100 nM control or *HELDR*-targeting ASO in vitro. **b,** IB for indicated proteins in GBM6 cells 72 hours after transfection with 100 nM control or *HELDR-targeting* ASO in vitro. **c, d,** cell proliferation (***c***), and glioma sphere formation (**d**) of GBM6 cells with indicated treatment. ASO, 100 nM; Erlotinib, 0.4 μM. **e,** The workflow of the animal experiment. **f,** BLI images, **g,** Kaplan-Meier analysis for mice bearing GBM6 brain tumor xenografts with indicated treatments. **h,** qRT-PCR analysis of *HELDR* expression in tumor area from brain sections. **i,** Graphical representation of the mechanism by which HELDR regulates EGFR treatment resistance and the translation strategy. **p < 0.01, ***p < 0.001, unpaired Student’s *t*-tests in **a,** one-way ANOVA in **c** and **h,** likelihood ratio test in **d,** log-rank test in **g**. Data are presented as mean ± s.d. (**a, c** and **h**).

Although blood-brain barrier (BBB) penetration and effective delivery are challenging, ASO treatment is still considered as a promising therapeutic approach for treating multiple neurological diseases^57^. Thus, we explored using ASOs targeting *HELDR* to treat GBM. ASO treatment significantly inhibited *HELDR* expression, the KAT7-H3K14ac/H4K12ac axis, and KAT7-regulated signaling pathways and gene expression as described above (Fig. 8a, b, Extended data Fig. 8a–c). Next, ASO treatment also enhanced Erlotinib suppression of GBM cell proliferation and self-renewal capacity in vitro (Fig. 8c, d, Extended data Fig. 8d, e). Last, we evaluated the therapeutic effects of individual or combination therapy by ASO and Erlotinib on orthotopic GBM6 tumor xenograft models (Fig. 8e). Both ASO and Erlotinib monotherapy suppress tumor growth with combination treatments showing significantly increased anti-tumor activity (Fig. 8f, g). Treatment with ASOs effectively suppressed *HELDR* expression in vivo (Fig. 8h), indicating on-target activity by ASO administration. In GBM xenografted tumors, ASO + Erlotinib treatment resulted in significantly lower proliferation indices (by Ki-67 staining) and higher apoptosis level (by cleaved caspase 3) comparing with monotherapy (Extended data Fig. 8f, g). In addition, both ASO and Erlotinib suppressed their known targets as well as targets identified in this study (Extended data Fig. 8f, g). Taken together, this data indicates ASO-targeting *HELDR* markedly enhanced anti-GBM activity of Erlotinib in orthotopic tumor xenografts in animals.

## Discussion

In this study, we identified and characterized a newly discovered IncRNA for its role in *EGFR*-driven GBM malignancy (Fig. 8i). This IncRNA that we name it *HELDR* is predominantly transcribed from the antisense strand of a previously reported lncRNA *ELDR* locus and is currently unannotated. *HELDR* can be co-amplified with *EGFR* within ecDNAs and promote GBM tumorigenesis independently of EGFR expression and signaling. *HELDR* globally regulates gene expression through its binding to DNA across the genome, frequently at gene promoters including *KAT7. HELDR* binds and recruits p300 to the KAT7 promoter, leading to increased H3K27ac, enhanced transcription factor binding, and activation of KAT7 transcription. Elevated KAT7 increases H3K14ac and H4K12ac, inducing gene expression in signaling pathways critical for GBM tumorigenesis. Last, targeting KAT7 with a specific inhibitor or *HELDR* with ASOs enhances anti-GBM activity of the EGFR inhibitor, Erlotinib, in orthotopic GBM tumor xenograft models.

LncRNAs have been recognized as a critical output of the genomes of complex organisms^18^. Extensive efforts have been made to identify and characterize lncRNAs for their functions in development and disease, including cancer^19^. Thus far, all the annotated lncRNAs have been identified based on the levels of their expression or association with cellular processes or diseases. However, our discovery of *HELDR* was unexpected. We initially aimed to identify upregulated lncRNAs that correlate with *EGFR* expression in GBM. We found that the previously characterized *ELDR* is the top lncRNA with the highest correlation with EGFR. Significantly, *ELDR* co-amplifies with *EGFR* in the same ecDNAs in GBM tumors^5,6^. Moreover, our in-depth RNA seq analyses revealed an unannotated lncRNA, *HELDR,* in the *ELDR* locus that is transcribed from its antisense strand with high efficiency. We showed that *HELDR* promotes *EGFR*-driven GBM tumorigenesis by inducing genes in oncogenic pathways critical for GBM malignancy via binding to and recruiting p300 to *KAT7* gene promoter independent of EGFR expression and signaling. These are the second *(ELDR)* and the third *(HELDR)* lncRNAs that co-amplify with the driver oncogene *EGFR* at ecDNAs in GBM. We recently reported that the lncRNA *LINC02283* co-amplifies with *PDGFRA* and enhances PDGFRA-mediated signaling and promotes GBM tumorigenesis^23^. ecDNA amplifications that harbor driver oncogenes such as *EGFR, MYC, MDM2,* and *CDK4* were detected in 17.1% of 39 human tumor subtypes, including GBM^24^. ecDNAs are also enriched with regulatory ecDNAs including promoters, enhancers, and lncRNAs^24,25^. Thus, our discovery of the unannotated *HELDR* that coamplifies with *EGFR* in GBM ecDNAs and is transcribed in the antisense strand of the *ELDR* locus suggests that additional lncRNAs could be found in tumor ecDNAs and in the antisense strand of known gene loci. Since amplified lncRNAs can promote driver oncogene-promoted tumorigenesis dependent^23^ or independent (this study) of the driver oncogenic signaling, investigation of these co-amplified annotated and unannotated lncRNAs^24,25^ such as *LINC02283* and *HELDR* in tumor ecDNAs would further advance our understanding of their roles in driver oncogene-promoted tumorigenesis and therapy resistance of human cancers.

The cellular localizations and interaction of a IncRNA with RNAs, DNAs, or proteins dictates its role in modulating chromatin architecture, gene transcription, RNA processing, phase separation, DNA damage repair, or other cellular processes^18^. LncRNAs can also exhibit their cis- or trans-regulatory functions through RNA-DNA interactions that activate or silence gene transcription^20^. For examples, metastasis-associated lung adenocarcinoma transcript 1 *(MALAT1*), the most studied lncRNA in cancer, interacts with gene bodies and enhancers to induce WNT1 ligands and the Serpin protease inhibitor SERPINB6B that enables metastatic reactivation via immune evasion in breast cancer models^58^. Nuclear paraspeckle assembly transcript 1 *(NEAT1*) associates with gene promoters of key epithelial transcription factors to sustain their expression during epidermal differentiation^59^. In this study, we show that *HELDR,* an unannotated and GBM-specific nuclear *lncRNA* interacts with genomic DNAs at various regulatory regions including 25% gene promoter sequences. Specifically, *HELDR* binds to the promoters of *KAT7, UBASH3B,* and *DEPDC1.* We show that *HELDR* binds and recruits p300, a transcription factor and a histone acetyltransferase, to the *KAT7* promoter thereby enriching H3K27ac at the *KAT7* promoter and activating *KAT7* transcription. The increased KAT7 in turn catalyzes H3K14ac and H3K12ac on its target gene promoters, facilitating transcriptions of oncogenic genes critical for GBM tumorigenesis and enhancing the response to anti-EGFR therapies. Moreover, our data reveal that *HELDR* is globally associated with the promoters of more than 1,000 genes, including 200 genes with altered expression, which may be critical for GBM tumorigenesis. Thus, the effects of *HELDR*-regulated transcriptions through binding to their gene promoters in GBM tumorigenesis and therapy responses warrant further investigation.

KAT7 is a member of the lysine acetyltransferase (KAT) family that primarily induces H3K14ac and H4K12ac at gene promoters through forming alternative complexes with different subunits, thus epigenetically regulating gene expression^16,60^. P300 (KAT3B, CBP) induces H2, H3, and H4 acetylation at various lysine (K) residues including H3K27ac at promoters and active enhancers^60^. A previous study used in vitro synthesized RNA fragments of lncRNA HOTAIR or HAT, rather than endogenous lncRNAs, to demonstrate that P300 acetyltransferase activity is stimulated by lncRNA binding^13^. Our data of KAT7 and p300 activities regulated by *HELDR* in GBM corroborates with these studies. We show that in *EGFR-*amplified GBM cells, endogenously co-amplified *HELDR* interacts with *KAT7* promoter and recruits p300 to its promoter. The stimulated p300 activity enriches H3K27ac at the *KAT7* promoter, thereby inducing KAT7-targeting genes that are critical for EGFR-driven GBM tumorigenesis and enhancing GBM response to anti-EGFR therapies. Together, our data reveals an uncharacterized mechanism by which co-amplified *HELDR* promotes *EGFR*-driven malignancy by stimulating p300 activity and inducing *KAT7* expression in GBM.

*EGFR* is a driver oncogene^4^ and a therapeutic target for treating GBM^8^. Thus far, anti-EGFR treatments have been disappointing largely due to tumor heterogeneity, redundant downstream signaling and an immune-suppressive tumor microenvironment in GBM^9^. In this study, we reveal an uncharacterized *HELDR-KAT7* axis that promotes *EGFR*-driven GBM tumorigenesis and therapy resistance independent of EGFR expression and signaling. Our in vitro tests show that targeting KAT7 by a specific inhibitor WM-3835^61^ or *HLEDR* by ASOs synergistically enhanced cytotoxicity of the EGFR inhibitor Erlotinib in GBM cells. In vivo, we also show that treatments with WM-3835 or ASO in combination with Erlotinib displayed markedly enhanced anti-GBM activity and significantly prolonged overall survival of GBM tumor xenograft-bearing animals. Our data that targeting *HELDR*-activated KAT7 or targeting *HELDR* by ASOs augments anti-GBM activity by Erlotinib validated our observation that the previously uncharacterized *HELDR-KAT7* axis promotes *EGFR*-driven GBM tumor malignancy independent of EGFR signaling.

In summary, this study reports the role of the unannotated lncRNA *HELDR* that can be co-amplified with *EGFR* within ecDNAs in GBM and contributes to tumorigenicity and therapy response. We show an undescribed *HELDR-KAT7* axis that enhances GBM tumor malignancy independent of EGFR signaling. The combination therapy, specifically targeting the *HELDR*-induced KAT7 activity or *HELDR-ASO* enhances cytotoxicity of Erlotinib for treating orthotopic GBM tumor xenografts in animals. This study could have a significant impact on advancing our understanding of the tumor biology of oncogene-driven GBM and other human cancers, through investigating the roles of annotated and unannotated lncRNAs within ecDNAs in GBM and other cancers.

## Materials and methods

### Cell culture

Human HEK293T cells, U87 cells, and GBM6^29^ cells were cultured in DMEM (Thermo Fisher Scientific, 11995–065) supplemented with 10% FBS (Thermo Fisher Scientific, 10437028) and 1% penicillin-streptomycin (Thermo Fisher Scientific, 15140122). Patient-derived GSCs including GSC157^23^, GSC7–2, GSC17, GSC23, GSC11, GSC34^62^, and GSC1478^35^ were cultured in GSC medium. The GSC medium consists of DMEM/F12 medium (Thermo Fisher Scientific, 11320–033), 2% B27 supplement (Thermo Fisher Scientific, 17504–044), 1X antibiotic-antimycotic (Thermo Fisher Scientific, 15240062), 5 mg/mL heparin (Sigma-Aldrich, 9041–08-1), 20 ng/mL EGF (Peprotech, 100–15R), and 20 ng/mL bFGF (Peprotech, 100–18B). All cells were authenticated by short tandem repeat analysis at IDEXX BioAnalytics, Texas Tech University Health Sciences Center (Lubbock, TX), or Northwestern University’s NUSeq core facility. All cell lines tested negative for Mycoplasma using the VenorGeM Mycoplasma Detection Kit (Sigma-Aldrich, MP0025). The latest authentication and Mycoplasma testing were performed in December 2022. All cell lines were cultured for fewer than 20 passages prior to use.

### RNA extraction and purification

RNA extraction and purification were conducted using the RNeasy Mini Kit (Qiagen, 74104) according to the manufacturer’s instructions. Under some situation, we combine the TRIzol (Invitrogen, 15596026) method with the RNeasy Mini kit to purify RNA.

### PCR, qPCR and qRT-PCR

cDNA was generated via reverse transcription using the iScript cDNA Synthesis Kit (Bio-Rad, 1708891) according to the manufacturer’s instructions. PCR was performed using the Q5 High-Fidelity PCR Kit (NEB, E0555S) according to the manufacturer’s instructions. EvaGreen qPCR MasterMix (Bullseye, BEQPCR-R) on an Applied Biosystems StepOne Plus Real-Time Thermal Cycling Block was used to conduct qPCR and qRT-PCR. The relative level of gene expression was calculated using the ΔΔCt method. Indicated primers are shown in Supplementary Table 9.

### Subcellular fractionation

Cells were lysed in ice-cold PBS/0.1% NP-40. After centrifugation at 720 xg for 5 min, the supernatant was collected as the cytoplasmic fraction. The pellet (nuclear fraction) was washed and dissolved in either SDS lysis buffer (for protein) or TRIzol (for RNA), depending on the intended application.

### RACE

RACE was conducted using the SMARTer^®^ RACE 5’/3’ Kit (Takara, 634859) according to the manufacturer’s instructions. Briefly, 1 μg of freshly isolated RNA was used to generate first-strand cDNA. 5’ and 3’ RACE PCRs were performed with universal and gene-specific primers listed in Supplementary Table 9. For 5’ RACE PCR, the following conditions were used: 94°C for 30 sec, 68°C for 30 sec, and 72°C for 3 min, for a total of 30 cycles. For 3’ RACE PCR, a nested PCR strategy was employed. Specifically, the first round of PCR was performed with the following conditions: 94°C for 30 sec, 68°C for 30 sec, and 72°C for 3 min, for 30 cycles. The second round of PCR used these conditions: 94°C for 30 sec, 70°C for 30 sec, and 72°C for 3 min, for 25 cycles. PCR products were visualized by agarose gel electrophoresis and cloned into a linearized pRACE vector. We randomly selected 12 clones for Sanger sequencing for both 5’ RACE and 3’ RACE.

### Single-molecule RNA fluorescence in situ hybridization (smFISH) and immunofluorescence (IF) staining

Cells were grown on coverslips. Cells or frozen xenograft tissue sections were fixed with 3.7% formaldehyde in 1X PBS at room temperature for 10 min.

For smFISH, fixed cells were washed with 1X PBS and then permeabilized in 70% ethanol at 4°C overnight. On day 2, a probe pool was diluted in a hybridization solution containing 10% formamide, 2X saline sodium citrate (SSC), and 10% dextran sulfate (w/v), with a final concentration of 1–10 nM of each probe. The 3’-biotinylated probe pool used was the same as in the ChIRP assay. Hybridization was conducted overnight in a humid chamber at 37°C. Cells were then washed twice at 37°C for 30 min with 10% formamide in 2X SSC. Afterward, the cells were incubated with Alexa Fluor 594-conjugated streptavidin (Thermo, S32356) and DAPI (Invitrogen, 2615842) for 20 min at room temperature. Finally, the cells were rinsed twice with 2X SSC. *HELDR*-KO cells and RNase-treated cells were used as negative controls. In RNase treatment control, cells were treated with RNase A (NEB, T3018L) at 37°C for 30 min prior to the hybridization step.

For IF staining for cells, fixed cells were washed and permeabilized with 0.5% Triton-X 100 at 4°C for 5 min. Then, the cells were incubated with the indicated antibody (Supplementary Table 10) at room temperature for 1 h. For fixed frozen xenograft tissue sections, the tissues were incubated with the indicated antibodies at 4°C overnight. After washing with 1X PBS, coverslips or slides were incubated with the appropriate fluorophore-conjugated antibody (Supplementary Table 10) and DAPI (Invitrogen, 2615842) at room temperature for 20 min. After washing, coverslips or slides were mounted with Fluoro-Gel (Electron Microscopy Sciences, 17983–20). The images were acquired using an Olympus BX53 microscope equipped with a DP72 camera.

### Cell viability assay

Cells were seeded at a density of 1,000 cells per well with triplicates in a 96-well plate. The CellTiter-Glo Luminescent Cell Viability Assay Kit (Promega, G9241) was used to determine cell viability following the manufacturer’s instructions. Luminescence was measured using the SpectraMax M3 Multi-Mode Microplate Reader (Molecular Devices).

### Limiting dilution assays

Single-cell suspension of GSCs was seeded into 96-well plates with varying cell numbers. After one week of culture, the wells with tumor sphere formation were counted, and the results were calculated using the Extreme Limiting Dilution Analysis (ELDA) online tool (http://bioinf.wehi.edu.au/software/elda/).

### RNA pull-down assay

DNA templates for in vitro transcription were acquired by PCR reaction. Biotinylated RNAs were generated using an in vitro transcription kit (Thermo, A57622) according to the instructions. DNA templates were then removed by digestion with DNase I (NEB, M0303L) at 37°C for 30 min. RNAs were purified using the RNeasy Mini Kit (Qiagen, 74104).

In vitro transcribed RNA was heated to 65°C for 10 min, then slowly cooled to room temperature to allow proper secondary structure formation. GSC cells were lysed in RIPA buffer (50 mM Tris, pH 7.4, 150 mM NaCl, 1 mM EDTA, 0.1% SDS, 1% NP-40, 0.5% sodium deoxycholate, 0.5 mM DTT, 1X PIC, and 1X PhIC). The cell lysate was mixed with RNA and incubated at 4°C for 4 h on a rotor. The reaction mixture was then incubated with streptavidin-conjugated magnetic beads (Thermo, 65001) at 4°C for 1 h on a rotor. The beads were washed five times with RIPA buffer. RNA-associated proteins were eluted by boiling in SDS buffer and detected by Western blot.

### RNA immunoprecipitation (RIP)

Cells were crosslinked with 0.3% formaldehyde at room temperature for 10 min. To neutralize the formaldehyde, 1.25 M glycine was added to a final concentration of 0.125 M, and the mixture was incubated at room temperature for 5 min. Cells were then washed twice with PBS and lysed in RIPA buffer. The cell lysate was mixed with an antibody and incubated at 4°C for 4 h on a rotor. The reaction mixture was subsequently incubated with protein A agarose beads (CST, 9863) at 4°C for 1 h on a rotor. The beads were washed ten times with RIPA buffer. Proteinase K (NEB, P8107S) and reaction buffer were added, and the mixture was incubated for at 55°C for 30 min. The RNA was retrieved as described in the RNA extraction protocol.

### CRISPR/Cas9-mediated HELDR deletion

The gRNAs were designed using the SYNTHGO CRISPR Design Tool for knockouts (https://design.synthego.com). Two gRNAs were cloned into the lentiCRISPRv2GFP plasmid (Addgene, 82416)^63^, and two gRNAs were cloned into the lentiCRISPR v2 plasmid (Addgene, 52 961)^64^. Plasmids for sequence-verified clones were co-transfected into HEK293T cells with the packaging vector psPAX2 (Addgene, 12260) and pVSV-G (Addgene, 138 4 79)^65^ to produce lentiviral particles. GSC cells were infected with the virus overnight and then selected using puromycin (Thermo Fisher Scientific, A1113803) and the FACSMelody 3-Laser Sorter (BD Biosciences). Single clones were selected and genotyped by PCR and Sanger sequencing.

### Gene overexpression and knockdown

All the relevant plasmids were acquired from VectorBuilder. Sequences were incorporated into PiggyBac expression vectors, whereas the shRNA target sequences were inserted into U6-based PiggyBac shRNA vectors. The PiggyBac plasmid was co-electroporated with the PiggyBac Hypase Plasmid using the Neon Transfection System (Invitrogen, MPK10096) as per the manufacturer’s instructions. The information of indicated plasmids are shown in Supplementary Table 11.

### Immunoblotting (IB)

Cells were lysed using an SDS lysis buffer consisting of 2% SDS, 50 mM Tris-HCl, 10 mM EDTA, and 10% glycerol (pH 8), supplemented with 1X protease inhibitor cocktail (Sigma-Aldrich, P8340) and 1X phosphatase inhibitor cocktail (Roche, 4906845001). The protein samples were then separated via SDS-PAGE and transferred to nitrocellulose (NC) membranes. After the transfer, the membranes were blocked with 5% non-fat milk in TBS-T at room temperature for 1 h. They were then incubated with the indicated antibodies at 4°C overnight. On the second day, following washing with TBS-T, the membranes were incubated with host-specific secondary antibodies conjugated with horseradish peroxidase (HRP). Signal was developed using enhanced chemiluminescence (ECL) (Amersham Bioscience, RPN2109) reaction according to the manufacturer’s instructions. The image of ECL signals on IB membranes were captured by using an iBright CL1500 Western Blot Imaging System (ThermoFisher).

### Short-read RNA sequencing

Libraries were prepared using the NEBNext RNA Ultra Library Preparation Kit (E7770) with the poly(A) enrichment module for total RNA. The libraries were sequenced on a NOVAX-02_NovaSeq X Plus (Illumina) at Northwestern University’s NUSeq core facility, resulting in an average coverage of 100 million 150 bp paired-end reads per sample. Sequencing data were processed using Quest, Northwestern University’s High-Performance Computing Cluster. Reads were aligned to the human genome reference hg38 using HISAT2. Differential gene expression analysis was performed using the DESeq2 package in R software. Gene enrichment analysis was conducted using the online software ShinyGO (http://bioinformatics.sdstate.edu/go/). GSEA analysis was performed using the fgsea package in R software.

### Long-read RNA sequencing

The long-read RNA-seq was performed using the platform from Oxford Nanopore Technologies (ONT). Poly(A) RNA was purified from the total RNA sample using the DynaBeads mRNA Purification Kit (Invitrogen, 61006). The SQK-DCS109 kit (ONT) was used to prepare the library according to the manufacturer’s instructions. Sequencing was conducted on a Nanopore MinlON platform at Northwestern University’s NUSeq Core Facility. ONT reads were base-called and demultiplexed using Guppy. The sequencing data was analyzed using Porechop and FLAIR according to the tools’ standard workflows.

### ChIRP and ChIRP-seq

Twenty million cells per group were cross-linked with 1% glutaraldehyde in PBS at room temperature for 10 min. The cross-linking reaction was then quenched with 125 mM glycine at room temperature for 5 min. After centrifugation, the cell pellet was washed with cold PBS. Cells were lysed in lysis buffer (50 mM Tris-HCl, pH 7.0, 10 mM EDTA, 1% SDS) at a ratio of 1 ml per 100 mg of cells, with freshly added PMSF (Sigma-Aldrich, 10837091001), protease inhibitor cocktail (Sigma-Aldrich, P8340), and RNase inhibitor (Applied Biosystems, N8080119). The samples were then fragmented using a Q500 sonicator (Thermo). We designed biotinylated antisense oligo probes tiling HELDR, with sequences shown in Supplementary Table 12. Probes were numbered and then mixed in equimolar amounts into two independent pools: one containing even-numbered probes and the other containing odd-numbered probes. The hybridization reaction was conducted by adding two volumes of hybridization buffer (750 mM NaCl, 1% SDS, 50 mM Tris-HCl, pH 7.0, 1 mM EDTA, 15% formamide) with freshly added PMSF, protease inhibitor cocktail, and RNase inhibitor, to one volume of samples and 100 pmole of biotinylated DNA probe pools. The complex was pulled down using 100 μL of Streptavidin-magnetic C1 beads (Thermo, 65001) and washed five times with washing buffer (2x SSC, 0.5% SDS). For RNA isolation, 5 μL of Proteinase K (NEB, P8107S) and 95 μL of Proteinase K buffer (100 mM NaCl, 10 mM Tris-HCl pH 7.5, 1 mM EDTA, 0.5% SDS) were added and incubated at 50°C for 45 min with shaking. RNA was retrieved by combining TRIzol and the RNeasy Mini Kit. qRT-PCR was used to validate the specificity of the reaction.

For DNA isolation, DNA was eluted using 1 ml of elution buffer (50 mM NaHCO3, 1% SDS) containing 10 μL of RNase A (10 mg/mL) (NEB, T3018L) and 10 μL of RNase H (10 U/μL) (NEB, M0297L) at 37°C for 1 h with shaking. Next, 15 μL of Proteinase K (NEB, P8107S) was added to each sample, followed by incubation at 50°C for 45 min with shaking. The supernatant was then collected after separation by magnetic stand. The DNA was purified using the ChIP DNA Clean and Concentrator Kit (Zymo Research, D5205) according to the manufacturer’s protocol. The library preparation was performed using the KAPA HyperPrep Kit (Roche, 07962347001) and xGen UDI-UMI adapters (IDT, 10006914) following the manufacturer’s instructions. Subsequently, 150 bp pair-end sequencing was conducted using the Element AVITI sequencer (Element Biosciences) at Northwestern University’s NUSeq core facility.

### Analysis of ChIRP-seq

For ChIRP-seq data analysis, the paired-end sequencing reads were trimmed using fastp v0.22.0 and filtered using RNAnue v0.2.0. We applied window trimming (--wsize 3) with a minimum Phred score of 20. Quality control of the reads before and after preprocessing was conducted using FastQC v0.12.1. We aligned the pre-processed reads using Bowtie2 v2.5.1 with the --very-sensitive parameter against the GENCODE human release 45. Subsequently, the aligned reads were deduplicated using UMI tools v1.1.4. The bamCompare from deepTools v3.5.1 was used to to normalize the odd/even samples against the input samples. Reads Per Kilobase Million (RPKM) was also normalized. The resulting BigWig files were converted to bedgraph format by using bigWigToBedGraph from the standalone tools of the UCSC genome browser. Subsequently, the files were combined by using the custom script bedGraphOverlaps.py, which reports overlapping intervals and sums up the corresponding scores. Peaks were called for each replicate individually using MACS v3.0.1 with parameters --pvalue 0.05 --broad --broad-cutoff 0.05. Motif analysis was conducted using HOMER v4.11.

### ChIP and ChIP-re-ChIP

ChIP assays were performed by using the SimpleChIP^®^ Enzymatic Chromatin IP Kit (CST, 9003) according to the manufacturer’s instructions. Briefly, cells were crosslinked with 1% formaldehyde at room temperature for 10 min and then quenched with a glycine solution provided by kit. The crosslinked chromatin was digested with micrococcal nuclease and then sonicated using a Bioruptor (diagenode). The reaction system was incubated with the indicated antibody overnight at 4°C. After a 2-h incubation with Protein G beads at 4°C, the complex was de-crosslinked. The DNA was purified and subsequently used for qPCR.

For ChIP-re-ChIP, following the first round of immunoprecipitation with P300, H3K27ac, or IgG, the complex was eluted using 100 μL of elution buffer containing 10 mM dithiothreitol at 37°C for 30 min. The elution was diluted 25-fold with a buffer containing 20 mM Tris-HCl (pH 8.0), 150 mM NaCl, 2 mM EDTA, and 1% Triton X-100, followed by re-immunoprecipitation using the GABPA antibody.

### Animal experiments

All experiments were conducted under the Institutional Animal Care and Use Committee approved protocols at Northwestern University, in accordance with NIH and institutional guidelines. Athymic mice (Ncr nu/nu), aged 6 to 8 weeks, were purchased from Taconic Farms (Germantown, NY). For the in vivo phenotype experiments, GSCs or GBM6 cells expressing luciferase were injected intracranially with 2 X 105 cells. Bioluminescence imaging (BLI) was conducted to monitor tumor growth using the SII Lago imaging system (Spectral Instruments Imaging). In the therapy experiments, mice were injected intracranially with 2 X 10^5^ GSCs or GBM6-PDX cells. In the therapy experiment targeting KAT7, one-week post-implantation, the mice were randomly assigned to four groups: (I) vehicle; (II) WM-3835 (MCE, HY-134901) (100 mg/kg/day, Monday to Friday until moribund); (III) erlotinib (MCE, HY-50896) (50 mg/kg/day, Monday to Friday until moribund); and (IV) a combination of WM-3835 and erlotinib. In the therapy experiment targeting *HELDR,* one-week post-implantation, the mice were randomly assigned to four groups: (I) vehicle; (II) ASO targeting *HELDR* (4 μg/mouse/day, Monday and Thursday until moribund); (III) erlotinib (MCE, HY-50896) (50 mg/kg/day, Monday to Friday until moribund); and (IV) a combination of ASO targeting *HELDR* and erlotinib. For Kaplan-Meier survival analyses, mice were sacrificed when tumor-related symptoms appeared.

### Bioinformatics analysis of public datasets

To study the EGFR-correlated lncRNA expression profile in GBM, RNA-seq data was downloaded from the TCGA and CPTAC databases. Spearman correlation analysis between the expression of EGFR and each lncRNA was performed. lncRNAs with an absolute correlation coefficient (|r|) greater than 0.3 and a p-value less than 0.05 were included. A heatmap showing the expression of lncRNAs was generated with R software.

The RNA-seq data for GBM39, a PDX model with EGFR amplification, was obtained from a previous publication^5^. The data analysis pipeline was the same as that used for our in-house RNA-seq data.

Whole genome sequencing (WGS) data was used for copy number variation (CNV) analysis. Binned coverage on chromosome 7, encompassing the EGFR and HELDR genes, was calculated using the WGD suite (https://github.com/RCollins13/WGD). Three representative samples were visualized using the Sushi R package, version 1.34.0.

### Statistics

All grouped data are presented as mean ± SD. Comparisons between groups were performed using one-way ANOVA. Survival curves were estimated and plotted using Kaplan-Meier analysis, and differences between curves were compared using log-rank tests. Statistical analyses were conducted using GraphPad Prism, version 7.

## Supplementary Material

Supplement 1

Supplementary Tables 1 to 9 are not available with this version.

## Data Availability

The short-read RNA-seq, long-read RNA-seq, and ChIRP-seq data generated in this study have been deposited in the NCBI Gene Expression Omnibus (GEO) database under the accession number GSE286179, GSE286318, GSE286319, GSE286320, and GSE28643.
