# Supplementary material for: An EGFR Co-Amplified and De Novo Long Noncoding RNA HELDR Promotes Glioblastoma Malignancy through KAT7-Driven Gene Programs
Source: Res Sq. 2025 Jun 24:rs.3.rs-6456987. Preprint. [Version 1] doi: 10.21203/rs.3.rs-6456987/v1 (PMC12270211; doi:10.21203/rs.3.rs-6456987/v1)
Supplement: Supplement 1 [file NIHPPrs6456987v1-supplement-1.pdf]

Supplementary Files

This is a list of supplementary files associated with this preprint. Click to download.

- Extendedfigures.docx
